# A semi-competing risks model for data with interval-censoring and informative observation: An application to the MRC cognitive function and ageing study

**DOI:** 10.1002/sim.4071

**Published:** 2010-11-06

**Authors:** Jessica K Barrett, Fotios Siannis, Vern T Farewell

**Affiliations:** a MRC Biostatistics UnitCambridge, U.K.; b Department of Mathematics, University of AthensGreece

**Keywords:** multi-state models, semi-competing risks, Weibull models, interval-censored data

## Abstract

Semi-competing risks data occur frequently in medical research when interest is in simultaneous modelling of two or more processes, one of which may censor the others. We consider the analysis of semi-competing risks data in the presence of interval-censoring and informative loss-to-followup. The work is motivated by a data set from the MRC UK Cognitive Function and Ageing Study, which we use to model two processes, cognitive impairment and death. Analysis is carried out using a multi-state model, which is an extension of that used by Siannis *et al*. (*Statist. Med.* 2007; **26**:426–442) to model semi-competing risks data with exact transition times, to data which is interval-censored. Model parameters are estimated using maximum likelihood. The role of a sensitivity parameter *k*, which influences the nature of informative censoring, is explored. Copyright © 2010 John Wiley & Sons, Ltd.

## 1. Introduction

A competing risks framework consists of survival data where failure may be due to one of a number of competing causes. This notion can be extended to that of semi-competing risks, where one type of event may censor the other events, but not vice versa. The ‘censoring’ event is sometimes known as the terminal event. A simple example of a semi-competing risks model is an illness-death model, where death may occur after illness, but death censors illness. In this case individuals who have a greater risk of death may also have a greater risk of illness, resulting in informative censoring of illness by death. Semi-competing risks frameworks have previously been discussed in Day *et al.* 1 and Fine *et al.* 2. A multi-state model approach to semi-competing risks data was suggested by Wang 3. We will use a fully parametric version of multi-state models reviewed by Putter *et al.* in 4, extended to allow for interval censoring. Covariates are incorporated naturally via proportional hazards assumptions.

Our work is motivated by the MRC UK Cognitive Function and Ageing Study (MRC CFAS), which is a longitudinal study investigating the cognitive function of older people 5. Cognitive function has been assessed in this study using a continuous measure, but for research purposes has been dichotomized into two states: healthy and cognitively impaired (CI). Our aim is to jointly model two processes, CI and death. The CI process may be censored informatively by death, but there is an additional censoring process which may also be informative—participants who are CI may be more likely to withdraw from the study. We will therefore consider a semi-competing risks model with a death process, and two competing non-terminal processes, CI and loss-to-followup (LTF). A multi-state model analysis of the MRC CFAS data that does not take account of informative LTF has been carried out by van den Hout and Matthews 6.

Our model extends that of Siannis *et al.* 7, who consider a five-state model where the LTF process is modelled explicitly by introducing LTF states in the illness-death model. Siannis *et al.* 7 assumed that transition times were known exactly. The situation is more complicated for the analysis of the MRC CFAS data because there are long gaps of up to 8 years between measurements of cognitive function; hence, we must consider the data to be interval-censored. As a result our model will require an additional transition compared with the model used by Siannis *et al.*

In Section 2 we will describe the MRC CFAS. In Section 3 we will describe our model and methods of inference. In Section 4 we will present our data analysis, and inSection 5 we give some concluding remarks and discuss outstanding issues.

## 2. MRC CFAS

MRC CFAS is a UK-based longitudinal multi-centre study investigating the health and cognitive function of older people 5. The six centres in the study are Newcastle, Nottingham, Liverpool, Cambridgeshire, Gwynedd and Oxford. CI was assessed using the Mini-Mental State Examination (MMSE), a widely used test of memory and cognitive function 8. MMSE scores range from 0 to 30 with scores less than or equal to 21 indicating CI. Participants who were assessed as CI at the prevalence screen, or at the incidence screen 2 years later, were scheduled to have assessments every one to 3 years. A random sample of healthy participants also followed this assessment schedule. The remainder were assessed less frequently, with gaps of up to 8 years between assessments, which means that we must consider the data to be interval-censored. All participants were flagged in the Medical Research Information Service, so exact death times were recorded for all those who died during the study, including those who were LTF. Because the pattern of observations is fixed by the design of the study, it is not informative in MRC CFAS. In other contexts this may not be true, and might require consideration.

## 3. Methods

### 3.1. Semi-competing risks: the model

**Figure 1:**
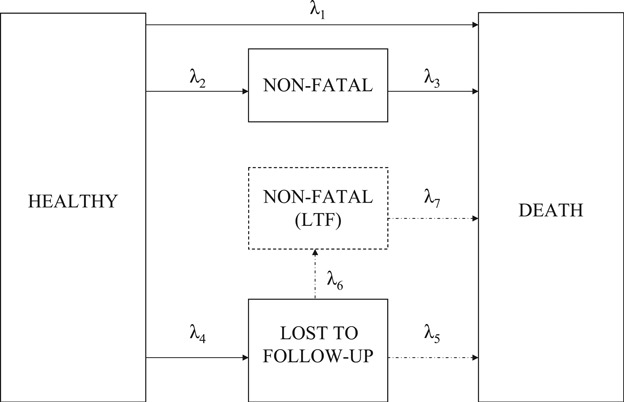
Five-state model for data from the Whitehall study.

Figure 1 shows the model used by Siannis *et al.* 7 to model cardiovascular disease with data from the Whitehall study. In this model the process of being LTF is modelled explicitly by the inclusion of an LTF state in the model. Assumption of non-informative LTF has been avoided by allowing transition rates to a Non-Fatal event and death to differ after LTF (i.e. λ_1_≠λ_5_ and λ_2_≠λ_6_). Non-fatal events were not observed after LTF, hence, Non-Fatal(LTF) is an unobserved state, indicated in the model diagram by a dotted box. There is no transition from the Non-Fatal state to the LTF state in this model because the interest is in the time of the first non-fatal event, after which LTF provides no extra information as full information is available on mortality.

**Figure 2:**
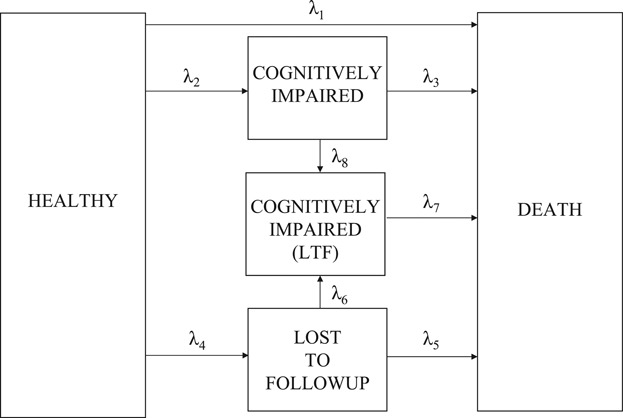
Five-state model for data from MRC CFAS.

Figure 2 shows the extension of this model that we have used to model the interval-censored data from MRC CFAS. In this model CI takes the place of the Non-Fatal state in the Whitehall model. We have introduced a transition from the CI state to the LTF state because otherwise participants who become LTF are assumed to be healthy until the time of LTF. This was not a problem for the Whitehall data because exact transition times were assumed to be known, and so any non-fatal event prior to LTF would have been observed. For the interval-censored data set we can no longer make this assumption, and we must therefore allow the possibility of participants becoming CI in the time interval between the last healthy observation and the time of LTF. The state Cognitively Impaired And Lost-To-Followup (CI(LTF)) is now observed for participants LTF after CI. But it remains unobserved for those who were healthy prior to LTF because we do not know whether or not they become CI later on. In this model we have implicitly assumed that the hazard of death for those who are in the CI(LTF) state is independent of the order in which CI and LTF has occurred.

### 3.2. The likelihood

There are 12 routes that we might observe a participant to take in the MRC CFAS model. They are (1) H→H, (2) H→CI, (3) H→CI→D, (4) H→D, (5) H→LTF, (6) H→LTF→D, (7) CI→CI, (8) CI→D, (9) H→CI→CI(LTF), (10) H→CI→CI(LTF)→D, (11) CI→CI(LTF) and (12) CI→CI(LTF)→D, where ‘H’ represents the healthy state, ‘CI’ is the cognitively impaired state, ‘D’ is the death state, ‘LTF’ is the lost-to-followup state and ‘CI(LTF)’ is the cognitively impaired and lost-to-followup state. Some observable routes correspond to more than one model trajectory, for example observable route (5) corresponds to H→LTF and H→LTF→CI(LTF).

We will fit the model using maximum likelihood estimation. The likelihood function is a product of the probabilities of observed routes


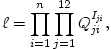


where *Q*_*ji*_ is the probability of observing route *j* for subject *i* and *I*_*ji*_ is an indicator function for subject *i* taking route *j*. Expressions for the *Q*_*ji*_ are given in the Appendix. Because our data are interval-censored, we must sum over all possible trajectories that might have taken place between observations when calculating a transition probability. For example, if we observe a transition from the healthy state to the death state we must allow for the possibility of the participant having passed through the CI and/or LTF states between the last healthy observation and the time of death.

We take the timescale *t* to be the time from study entry, as in van den Hout and Matthews 6 (see also the discussion in Siannis *et al.* 7). We use Weibull hazards for transition rates:


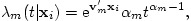
(1)

where α_*m*_ is the shape parameter for transition *m*, *m* = 1, …, 8, **x**_*i*_ is a vector of explanatory variables for subject *i* and **v**_*m*_ is a vector of explanatory variable coefficients for transition *m*. We again use age and sex as covariates. We use a logistic model for the probability of a participant being CI at the prevalence screen, again with age and sex as covariates, as was used by van den Hout and Matthews in 6.

### 3.3. Constraints

Not all of the parameters in the MRC CFAS model are identifiable, and we must therefore introduce some constraints on parameters. We use one constraint as used by Siannis *et al.*:


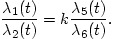
(2)

Although somewhat unusual, this constraint was chosen to impose minimal restrictions on parameter values. The assumption is that the ratio of the hazard of death to the hazard of CI for those who are not LTF is proportional to the equivalent ratio for those who are. Note that it is only the intercept parameters which are affected by the choice of *k* in equation (2). In particular, regression coefficients for explanatory variables should be robust to changes in the value of *k*. (We thank one of our referees for highlighting this.) There is no information in the data concerning the value of the parameter *k*, and this parameter must therefore be regarded as a fixed constant. We will test the sensitivity of our results using various values of *k*.

The sensitivity parameter *k* in equation (2) plays a very specific role in the analysis, exploring certain aspects of non-ignorable censoring. As a default, analysis is carried out assuming *k* = 1, which means that the ratio of the‘healthy to death’ rate to the CI rate is not affected by LTF. The assumption of *k* = 1 does not exclude the possibility of informative LTF, since the two rates may change in proportion to each other after LTF, even though the ratio remains unchanged. The parameter *k*, therefore, measures the relative change of one rate to the other following LTF. If *k*>1 then the CI rate increases more after LTF relative to the death rate, while *k*<1 implies exactly the opposite.

Unlike Siannis *et al.* we do not need to introduce any further parameter constraints to ensure identifiability of our model. This is a consequence of the assumption, discussed earlier, that the hazard of death for those who are CI and LTF does not depend on the order in which those events occurred. It is therefore possible, in principle, to estimate λ_7_ parameters using the observable data of those who became LTF after CI, because we can be certain that they entered the CI(LTF) state. However, to reduce the number of parameters in our model we impose the additional (but not required) constraint


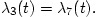
(3)

This constraint implies that the hazard of death for CI participants is independent of whether or not they are LTF. This assumption appears reasonable if we regard the critical information to be whether or not a participant is CI, and that CI has the same effect before or after a participant is LTF.

## 4. Data analysis

### 4.1. Data assumptions

For simplicity only data from the Newcastle centre have been used. The initial data set contained 2524 participants. We removed from this data set 56 who had missing MMSE scores at the prevalence screen, 4 who were audited as dead but with no record of the date of death and a further 12 participants, for whom our information was uncertain at the time of analysis. The data set used in the analysis therefore contained a total of 2452 participants.

In the MRC CFAS data set exact times of LTF have not been recorded, only the interview at which the participant was first LTF. Because interviews are scheduled at varied times, we have to define an interval during which LTF can be considered to have taken place. As an upper end-point for the LTF interval we could use the average time since prevalence screen of the missed interview. However, this can cause problems when interviews are close together because an interview may take place later than the average time of the interview scheduled to follow it. Instead we have used the time of the last interview which took place plus the average time to the next scheduled interview as an upper end-point for the LTF interval.

As described earlier, when calculating the probability of a transition in the likelihood, we must sum over all possible transitions which may have taken place during the intervening period. In practice this means that we never observe a direct healthy to death transition, because we must also allow for the probabilities of passing through the CI and/or LTF states between the last healthy observation and the time of death. We found that a likelihood composed in this way may lead to misleading parameter estimates because an unfeasible set of parameter values may have a high likelihood. For example a near-zero healthy-to-death transition rate may be compensated for by other death rates being inflated. To avoid this problem we introduced an extra assumption that participants who died within 2 years of their last observation did not become CI or LTF during those 2 years.

### 4.2. Results

The age range of participants in the data set was 64–103 years at baseline, with a median of 74 years and an inter-quartile range of 11 years. Of the 2452 participants in the data set, 908 were male and 1544 were female.

Before presenting the results of estimation of our multi-state model, we will first consider a simpler time-to-event analysis which may shed light on the relationship between the death, CI and LTF processes. Table II shows results of proportional hazards regression analyses for time to death with (a) CI and (b) LTF as time-dependent covariates, and with age in years and sex as time-independent covariates. This analysis assumes non-informative censoring. This assumption can be justified for the LTF analysis because censoring only occurs at the end of the study. It may not be justified for the CI analysis where individuals who are LTF have been censored at their last observation time. The results of analysis (a) show a considerable increase in risk of death for participants who were CI. The results of analysis (b) suggest that there may be a slight increase in the risk of death for participants who have become LTF. For both analyses the results show a higher risk of death for older ages and a lower risk of death for females, as we would expect.

**Table II:** Results of proportional hazards analyses for time to death with time-dependent covariates to indicate (a) CI and (b) LTF

Hazard ratio (95 per cent CI)			

	(a)		(b)
CI-Indic	2.048 (1.729 − 2.427)	LTF-Indic	1.105 (0.987 − 1.236)
Age	1.092 (1.081 − 1.103)	Age	1.098 (1.090 − 1.107)
*Sex*		*Sex*	
Male	1	Male	1
Female	0.676 (0.593 − 0.770)	Female	0.664 (0.596 − 0.738)

In the remainder of this section we will consider the multi-state model of Figure 2. Counts of observed transitions between states are given in Table I. We have fitted the model in R using maximum likelihood estimation. Table III shows maximum likelihood estimates for the model parameters. Here transitions have been divided into transitions to death and others. The parameters relating to λ_6_ and λ_7_ can be determined by the constraints (2) and (3). For the death transitions older participants and male participants have a greater hazard of death, as we would expect. The intercept and shape parameters of λ_3_ are greater than those of λ_1_ indicating a greater hazard of death for CI participants, which agrees with the results of the simple proportional hazards analyses. The λ_5_ intercept and shape estimates are somewhat different from the λ_1_ estimates, with a smaller hazard at earlier times, but a greater hazard at later times due to the larger shape parameter. For other transitions, the H→CI hazard is greater for older and female participants, whereas the H→LTF hazard is greater for female participants. The hazard of the H→LTF transition is decreasing with time (i.e. α_4_<1) because there was more drop-out from the study at earlier times. The CI→CI(LTF) transition has a very high intercept and small shape parameter because participants who were assessed as CI at the prevalence screen were scheduled to have a follow-up interview 1–2 months later, and there was a high level of drop-out before this second interview. It could be argued that participants who were CI at study entry could belong to a different population from those who were not. These individuals have been included partly because of the information they provide for estimation of the CI→CI(LTF) transition rate. Note, however, that if they are removed then the Weibull hazard for the CI→D transition is less elevated initially, and the hazard for the CI→CI(LTF) transition is smaller with more uncertainty about its shape.

**Table I:** Counts of observed events

	CI at *t* = 0	H → CI events	H → D events	CI → D events	H → LTF events	CI → LTF events	LTF → D events
Male	75	50	373	64	225	46	161
Female	213	134	421	182	499	130	335
Total	288	184	794	246	724	176	496

**Table III:** Maximum likelihood estimates for multi-state model parameters, standard errors are shown in brackets

	λ_1_ H→D	λ_3_ CI→D	λ_5_ LTF→D
*Death Transition Parameters*:			
Intercept	0.052(0.005)	0.094(0.015)	0.024(0.007)
Age	0.082(0.007)	0.053(0.007)	0.087(0.011)
Sex	−0.602(0.094)	−0.328(0.112)	−0.552(0.129)
Shape	1.156(0.045)	1.321(0.057)	1.636(0.117)
*Other Transitions*:			

	λ_2_ H→CI	λ_4_ H→LTF	λ_8_ CI→CI(LTF)
Intercept	0.013(0.002)	0.137(0.011)	1.710(0.774)
Age	0.133(0.011)	0.014(0.006)	−0.023(0.011)
Sex	0.455(0.162)	0.404 (0.081)	0.096(0.180)
Shape	1.163(0.072)	0.515(0.026)	0.092(0.044)
*Initial Distribution Parameters*:			
Intercept	2.524(0.129)		
Age	−1.342(0.100)		
Sex	−0.276(0.149)		

Table IV shows that the maximum likelihood estimates of the covariate parameters are fairly robust to different values of *k* (this was to be expected, as was discussed in Section 3.3). We have also fitted a *k* = 1 model with an age by sex interaction and a model with an age^2^ term in the linear predictor. There was little evidence that these additional variables improved the fit of the models.

**Table IV:** Maximum likelihood estimates for covariate parameters for different values of *k*

		Death transition covariates			Other transition covariates		
			
		λ_1_ H→D	λ_3_ CI→D	λ_5_ LTF→D	λ_2_ H→CI	λ_4_ H→LTF	λ_8_ CI→CI(LTF)
Age	*k* = 0.5	0.083	0.052	0.090	0.133	0.014	−0.023
	*k* = 1	0.082	0.053	0.087	0.133	0.014	−0.023
	*k* = 2	0.084	0.056	0.084	0.128	0.014	−0.023
Sex	*k* = 0.5	−0.561	−0.306	−0.535	0.437	0.404	0.092
	*k* = 1	−0.602	−0.328	−0.552	0.455	0.404	0.096
	*k* = 2	−0.636	−0.393	−0.518	0.460	0.412	0.098

In order to test for the presence of non-informative LTF in the data, we can compare the multi-state model with a model which is identical, except with λ_1_ = λ_5_ and λ_2_ = λ_6_. A likelihood ratio test gave a test statistic of 16.3 on 4 degrees of freedom, which corresponds to a *p*-value of 0.003. There is therefore evidence that the more complicated model provides a better fit to the data.

**Figure 3:**
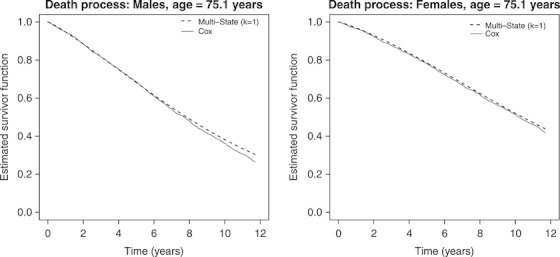
Comparison of survival curves for the death process from the non-parametric analysis with the multi-state model analysis.

The time to death process can be modelled non-parametrically using an adapted Kaplan–Meier estimate of the survival curve with adjustment for age and sex through the Cox model. This estimate should be unbiased because we have exact death times and no informative censoring for the death process. It can therefore be used to assess one aspect of the fit of the multi-state model by comparison with the multi-state estimate of the probability of survival. Figure 3 shows plots of the non-parametric and multi-state model survival curve estimates for males and females of mean study age. The multi-state model provides a generally reasonable fit, but with slight underestimation of the risk of death, especially for males at later times. This could be due to the extra assumptions made when fitting the multi-state model.

**Figure 4:**
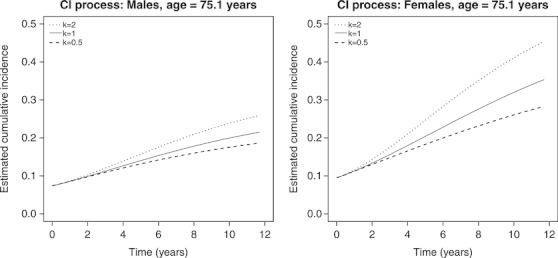
Cumulative incidence curves for the CI process using the multi-state model with various values of *k*.

We can also use the multi-state model to plot cumulative incidence curves for the time to CI outcome. Figure 4 shows plots of the cumulative incidence curves for the CI process, calculated using the multi-state model with various values of *k*. These plots are quite sensitive to the value of *k*, especially for females. The sensitivity to *k* appears to be greater here than was found in Siannis *et al.* 7 for the case with exact transition times, possibly because of extra uncertainty inherent in the use of interval-censored data.

## 5. Discussion

We have fitted a semi-competing risks model to data from the MRC Cognitive Function and Ageing Study, which takes into account informative LTF and interval-censoring. We used a five-state model, explicitly modelling the LTF process by the inclusion of two LTF states in the model. This paper extends previous work on MRC CFAS data 6 by allowing for informative observation. In addition, the presence of interval-censored data necessitated the use of a new model which has an extra transition compared with one used previously to handle informative observation 7. The results presented here agree qualitatively with those reported in the previous analysis. However, we have demonstrated that informative observation is present and therefore should be considered in future analyses of MRC CFAS data. In addition our analysis reveals that participants were more likely to become LTF from the MRC CFAS if they were older or female.

By using Weibull hazards for the transition intensities we were able to incorporate covariates in a natural way. However, other distributions could be used. If the model was to be used for prediction purposes, piecewise-constant intensities would be more appropriate 6.

The five-state model introduced here contains two LTF states and a transition between them which can never be observed. This leads to identifiability issues, and forces us to introduce constraints on some parameters in the model. One of the constraints we imposed involves a constant *k*, which is interpreted as the ratio of the hazard of death divided by the hazard of CI for those who are not LTF to the equivalent quantity for those who are LTF. Because there is no information about *k* in the data, it must be considered to be fixed in any estimation of the model. We used the most natural choice, *k* = 1, in our analysis, but also carried out sensitivity analyses using other values of *k*. We found the estimates of covariate parameters to be fairly robust to different values of *k*. However, estimated cumulative incidence curves were found to be more sensitive. The dependence of survival curves on *k* was more severe here than was found by Siannis *et al.* 7 using exact time transitions.

Our model could be extended in several ways. The assessment of cognitive function is subject to measurement error, and so we could take into account the possibility of misclassification of both healthy and CI states using methods similar to those of van den Hout and Matthews 6. Here we have concentrated instead on the time to the first assessment of CI and ignored later assessments, whether CI or not. Our approach can, however, be justified because in general participants show improvement in MMSE scores with time through familiarity with the MMSE questionnaire. The first CI assessment may therefore carry more weight, even though later assessments may indicate that the participant in question is healthy. Other possible extensions to our model could be to allow the hazard for the H→LTF transition to depend on the last recorded MMSE score, to allow for non-linear time effects, particularly for the H→CI transition, and to use a mixture model for the CI→CI(LTF) transition.

An alternative approach to the analysis of the MRC CFAS data set would be to use a joint longitudinal model in which the MMSE score was modelled as a continuous variable, and the hazard of death was allowed to depend on the MMSE score to account for the informative censoring of death by LTF due to cognitive decline. We used the multi-state model approach because the CI process itself is of clinical interest, as well as the death process, and we therefore wish to take into account informative LTF when modelling the CI process. In addition, modelling of the MMSE score as a continuous outcome variable is not straightforward (see, for example, Muniz Terrera *et al.* 9). Another advantage of the multi-state model is that it accounts for LTF which may be informative for a variety of reasons and for which relevant data may not be available to include as covariates in the model.

## Appendix A

In this appendix, we present expressions for the *Q*_*ji*_, the probability of subject *i* following route *j*. In these expressions all times are measured from study entry. We number the states as follows: Healthy is defined to be state 1, CI is state 2, Death is state 3, LTF is state 4 and CI(LTF) is state 5. The cumulative hazard functions *H*_*r*_(*t*_1_, *t*_2_) for leaving state *r* between time *t*_1_ and time *t*_2_ are

*f*_*i*_ is defined to be the probability of subject *i* being healthy at study entry, and is given by a logistic model with age and sex as covariates. Then the expressions for the *Q*_*ji*_ are:

1. H → H (with follow-up to time *t*_2_)
  (A1)
2. H → CI (last healthy observation at time *t*_2_, first CI observation at time *t*_3_ with follow-up to time *t*_4_)
  (A2)
3. H → CI → D (last healthy observation at time *t*_2_, first CI observation at time *t*_3_, last CI observation at time *t*_4_ with death at time *t*_6_)
  (A3)
4. H → D (last healthy observation at time *t*_2_ with death at time *t*_4_)
  (A4)
5. H → LTF (last healthy observation at time *t*_2_, LTF before *t*_3_ with follow-up to time *t*_4_)
  (A5)

where

6. H → LTF → D (last healthy observation at time *t*_2_, LTF before *t*_3_ with death at time *t*_4_)
  (A6)
7. CI → CI (with follow-up to time *t*_4_)
  (A7)
8. CI → D (with last CI observation at time *t*_4_ and death at time *t*_6_)
  (A8)
9. H → CI → CI(LTF) (last healthy observation at *t*_2_, first CI observation at *t*_3_, last CI observation at *t*_4_, LTF between *t*_4_ and *t*_5_ with follow-up till *t*_6_)
  (A9)
10. H → CI → CI(LTF) → D (last healthy observation at *t*_2_, first CI observation at *t*_3_, last CI observation at *t*_4_, LTF between *t*_4_ and *t*_5_ with death at *t*_6_)
  (A10)
11. CI → CI(LTF) (last CI observation at *t*_4_, LTF between *t*_4_ and *t*_5_ with follow-up till *t*_6_)
  (A11)
12. CI → CI(LTF) → D (last CI observation at *t*_4_, LTF between *t*_4_ and *t*_5_ with death at *t*_6_)
  (A12)
